# Fish Oil Supplementation Reduces Inflammation but Does Not Restore Renal Function and Klotho Expression in an Adenine-Induced CKD Model

**DOI:** 10.3390/nu10091283

**Published:** 2018-09-11

**Authors:** Juan S. Henao Agudelo, Leandro C. Baia, Milene S. Ormanji, Amandda R. P. Santos, Juliana R. Machado, Niels O. Saraiva Câmara, Gerjan J. Navis, Martin H. de Borst, Ita P. Heilberg

**Affiliations:** 1 Division of Nephrology, Federal University of São Paulo (UNIFESP), Rua Botucatu 740, 04023-900 São Paulo, Brazil; juanelmono17@hotmail.com (J.S.H.A.); leandronut@yahoo.com.br (L.C.B.); milene.ormanji@gmail.com (M.S.O.); amandda.rpds@gmail.com (A.R.P.S.); niels@icb.usp.br (N.O.S.C.); 2 Division of Nephrology, University of Groningen, University Medical Centre Groningen (UMCG), P.O. Box 30.001, 9700 RB Groningen, The Netherlands; g.j.navis@umcg.nl (G.J.N.); m.h.de.borst@umcg.nl (M.H.d.B.); 3 Tropical Medicine & Public Health, Federal University of Goiás (UFG), Rua 235 s/n-University Sector, 74605-050 Goiânia, Brazil; juliana.patologiageral@gmail.com; 4 Department of Immunology, Institute of Biomedical Sciences, University of São Paulo (USP), Av. Prof. Lineu Prestes 1730, ICB IV, Sala 238, 05508-000 São Paulo, Brazil

**Keywords:** klotho, CKD, fish oil, fibrosis, inflammation

## Abstract

Background: Chronic kidney disease and inflammation promote loss of Klotho expression. Given the well-established anti-inflammatory effects of omega-3 fatty acids, we aimed to investigate the effect of fish oil supplementation in a model of CKD. Methods: Male C57BL/6 mice received supplementation with an adenine-enriched diet (AD, *n* = 5) or standard diet (CTL, *n* = 5) for 10 days. Two other experimental groups were kept under the adenine diet for 10 days. Following adenine withdrawal on the 11th day, the animals returned to a standard diet supplemented with fish oil (Post AD-Fish oil, *n* = 9) or not (Post AD-CTL, *n* = 9) for an additional period of 7 days. Results: Adenine mice exhibited significantly higher mean serum urea, creatinine, and renal expression of the pro-inflammatory markers Interleukin-6 (IL-6), C-X-C motif chemokine 10 (CXCL10), and Interleukin-1β (IL-1β), in addition to prominent renal fibrosis and reduced renal Klotho gene expression compared to the control. Post AD-Fish oil animals demonstrated a significant reduction of IL-6, C-X-C motif chemokine 9 (CXCL9), and IL-1β compared to Post AD-CTL animals. However, serum creatinine, renal fibrosis, and Klotho were not significantly different in the fish oil-treated group. Furthermore, renal histomorphological changes such as tubular dilatation and interstitial infiltration persisted despite treatment. Conclusions: Fish oil supplementation reduced renal pro-inflammatory markers but was not able to restore renal function nor Klotho expression in an adenine-induced CKD model.

## 1. Introduction

Inflammation plays a central role in the pathogenesis and progression of chronic kidney disease (CKD). The activation of innate and adaptive arms of immune response leads to cell infiltration (mainly macrophages) and the production of proinflammatory molecules that ultimately lead to collagen deposition and loss of renal function [1]. 

The α-Klotho protein was originally identified as an anti-aging gene in 1997 and was later recognized as a transmembrane co-receptor of fibroblast growth factor (FGF23) [2]. Under healthy conditions, Klotho is a protein highly expressed in the renal distal convolute tubule [3], which under homeostatic conditions may also be present in soluble form in the blood, urine and cerebrospinal fluid. Soluble Klotho has several endocrine functions like anti-senescence, anti-oxidative, anti-renal angiotensin–aldosterone system (RAAS), and anti-inflammatory modulation [4]. Klotho deficiency is associated with reduced renal function, hyperphosphatemia, increased FGF23 levels, RAAS activation, and chronic complications such as ectopic calcification, cardiac hypertrophy, secondary hyperparathyroidism, and progression of CKD [4,5]. Klotho-deficient rodents exhibit manifestations of CKD and conversely, rodent CKD models show markedly reduced *Klotho* mRNA expression [4].The reasons why Klotho is reduced in patients with CKD are not completely understood, but it seems that inflammation could be one of the underlying mechanisms. The exogenous administration of TWEAK (Tumor Necrosis Factor-like weak inducer of apoptosis) decreased renal expression of Klotho and the blockade of TWEAK by neutralizing antibodies restored renal expression of Klotho [6]. These data suggest the existence of a bidirectional relationship between Klotho and inflammation. Therefore, treatment strategies targeting renal inflammation could potentially restore Klotho expression, reducing renal damage and preventing the associated comorbidities. 

Omega-3 fatty acids such as docosahexaenoic (DHA) and eicosapentaenoic (EPA) exert anti-inflammatory effects and may have reno-protective properties in kidney diseases [7,8]. Experimental data showed reduced tubulointerstitial cell infiltration, pro-inflammatory mediators such as Cyclooxygenase-2 (COX-2) and Monocyte chemoattractant protein-1 (MCP-1), and attenuation of fibrosis [9]. In addition, we previously observed that higher intake of EPA–DHA was independently associated with lower levels of FGF23 in renal transplant recipients [10], suggesting that omega-3 fatty acids could favorably affect the FGF23–Klotho axis. In the present study, we investigated whether fish oil, rich in omega-3 fatty acids, increases renal Klotho expression and reduces renal inflammation and fibrosis in a mouse model of inflammatory CKD. 

## 2. Materials and Methods 

### 2.1. Animal Model

**Figure 1:**
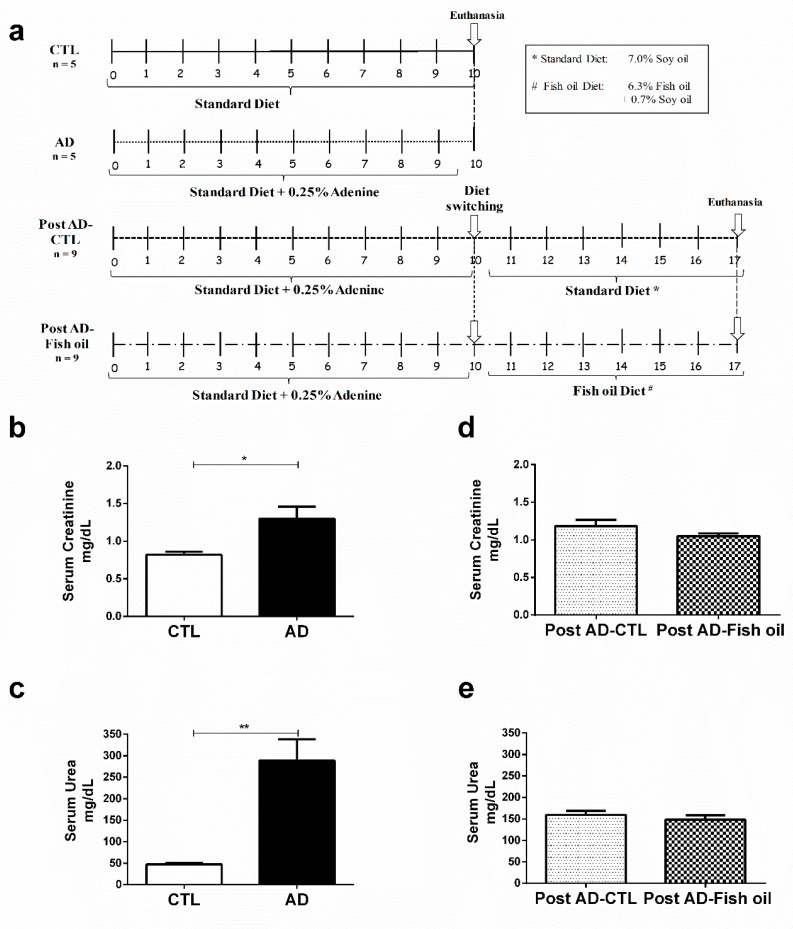
Experimental design and assessment of renal function: (**a**) experimental design, (**b**) serum creatinine, and (**c**) urea from the standard diet (CTL) and adenine (AD) mice. (**d**,**e**) Serum creatinine urea of Post AD-CTL and Post AD-Fish oil animals. (* *p* < 0.05, ** *p* < 0.01).

C57BL/6 wild-type mice, aged 8 to 12 weeks, were obtained from a local facility. All the procedures were developed according to international guidelines for care of laboratory animals and approved by the Animal Ethics Committee of the Federal University of São Paulo (CEUA, 1558280214). In order to ensure that renal inflammation and fibrosis were induced in this adenine CKD model, initial experiments were conducted over 10 days in two groups, which received either a standard diet (7.0% soy oil, CTL group, *n* = 5), or the same diet enriched with 0.25% adenine (AD group, *n* = 5). Once the renal inflammation and fibrosis were confirmed in the model, two additional experimental groups were initiated to evaluate the effects of fish oil supplementation. Both groups received adenine supplementation to the standard diet for 10 days. From the 11th day on, adenine administration was discontinued and the animals were either switched back to their standard diet (7.0% soy oil, Post AD-CTL group, *n* = 9), or started supplementation with fish oil (6.3%, Post AD-Fish oil group, *n* = 9), for 7 additional days (see experimental design in Figure 1a). The diets were purchased from Rhoster, Araçoiaba da Serra, Brazil and were in accordance with the American Institute of Nutrition recommendations (AIN 93G). At the end of the study, the animals were anesthetized with xylazine (10 mg/kg) and ketamine (50 mg/kg) by intra-peritoneal injection for blood sample collection by cardiac puncture and euthanized thereafter. The kidneys were harvested and immediately dissected, washed with saline, embedded in paraffin, sectioned longitudinally, and processed routinely for histologic examination. The remaining part was snap frozen in liquid nitrogen and stored at −80 °C. Serum creatinine was measured by the Jaffe modified method, and serum urea was measured using a Labtest Kit (Minas Gerais, Brazil) according to the manufacturer’s instructions. 

### 2.2. Real-Time PCR

IL-6 (Mm00446190_m1), IL-1β (Mm00434228_m1), TGF-β (Mm01178820_m1), HPRT (Mm00446968_m1), CXCL10, CXCL9, and *Klotho* gene expressions were assessed by real-time RT-Polymerase Chain Reaction (PCR). Renal tissues were crushed and homogenized, and the RNeasy Mini Purification Kit (Qiagen, Valencia, CA, USA) was used to extract RNA from all samples. RNA quantification was carried out using a NanoDrop Spectrophotometer (NanoDrop ND-1000 Spectrophotometer; Thermo Scientific, Wilmington, DE, USA). Nucleic acid concentration was determined using ultraviolet spectrophotometry at 260 nm and purity was determined using the absorbance ratios of 260/280 and 260/230. RNA was reverse transcribed using the QuantiTec SYBR Green Kit (Qiagen) and the manufacturer’s instructions were followed. Reverse transcription and Real-Time PCR were performed using commercially available reagents and a 7500 Fast Real-Time Thermocycler (Applied Biosystems, Carlsbad, CA, USA). For relative quantification of message expression (ΔCT Method), target gene expression was normalized to Hypoxanthine Phosphoribosyltransferase (*HPRT*) gene expression. 

The following primers were assessed for quantitative Syber Green RT-PCR:

KLOTHO fw: GGTGTCCATTGCCCTAAGCTC; KLOTHO rev: TCGGTCATTCTTCGAGGATTGA. CXCL9 fw: 5-TGCACGATGCTCCTGCA-3; CXCL9 rev:5-AGGTCTTTGAGGGATTTGTAGTGG-3. CXCL10 fw:5-GACGGTCCGCTGCAACTG-3; CXCL10 rev: 5-GCTTCCCTATGGCCCTCATT-3.

### 2.3. Histological Evaluation

Formaldehyde-fixed paraffin kidney sections (3 m) were dewaxed and stained with Picrosirius solution. Sections were immersed in saturated picric acid solution for 15 min and then in Picrosirius for 20 more minutes. Counter-staining was carried out with Harris hematoxylin. Picrosirius-stained sections were analyzed by an Olympus BX50 microscope (Olympus, Feasterville, PA, USA) with an Olympus camera attached. Manual shots were taken of the cortex, magnified 40X, and observed under polarized light. Photos of at least five different fields in each slide were taken, and structures such as the glomeruli, subcapsular cortex, large vessels, and medulla were excluded. The pictures were digitalized in a HP Scanjet 2400 (Hewllet Packard, Barueri, São Paulo, Brazil) and then the interstitial volume of collagen in the cortex compared to the overall cortex area was quantified by morphometry. For the morphometric analysis, the Image Processing and Analysis in Java (Image J—image processing program, National Institutes of Health, Maryland, MD, USA) was used. The result of the analysis is represented as a percentage of cortical interstitial collagen volume relative to the total cortical interstitial volume. Subsequently, the arithmetic mean of the analyzed fields was calculated for each slide. Collagen type I was associated with yellow/red birefringence and type III with green color, according to the description of Montes GS and Junqueira LC [11]. The assessment of the interstitial fibrosis volume obtained by morphometric analysis of the digital image stained with picrosirius was based on previous studies [12].

### 2.4. Western Blotting 

Here, 50 ug of total protein was obtained from kidney lysate of CTL, AD, Post AD-CTL, and Post AD-Fish oil mice. The protein extract was denatured by heating at 5 min at 95 °C and separated by 10% polyacrylamide gel electrophoresis (SDS-PAGE). Subsequently, protein extract was transferred into nitrocellulose membrane and next, the immunostaining was performed with the following primary antibodies: E-cadherin (Dako: M3612, Santa Clara, CA, USA), alpha smooth muscle actin (α-SMA, DAKO: M0851, Santa Clara, CA, USA), Klotho: (CosmoBIO: K0603, Carlsbad, CA, USA), and α-tubulin (InVitrogen: 32-2500, Sunnyvale, CA, USA). Then, nitrocellulose membrane was incubated with conjugated secondary antibodies (anti-rabbit or anti-mouse peroxidase/1: 125.0000, Sigma-Aldrich, St. Louis, MO, USA) and revealed by chemiluminescence methods using an ECL kit (Millipore, Burlington, MA, USA). Finally, the image was acquired on Amersham Imager 600 (GE Healthcare, Marlborough, MA, USA) and analyzed with Image J. 

### 2.5. Statistical Analyses

Differences between AD versus CTL and Post AD-CTL versus Post AD-Fish oil groups were assessed using Student’s *t*-test. All statistical analyses were performed using GraphPad Prism version 5.0 (GraphPad Software, La Jolla, CA, USA). The results are presented as mean and SD for parametric variables. Differences were considered significant if *p* < 0.05. 

## 3. Results

### 3.1. Adenine Supplementation Induces Inflammation, Loss of Renal Function, and Klotho Reduction

**Figure 2:**
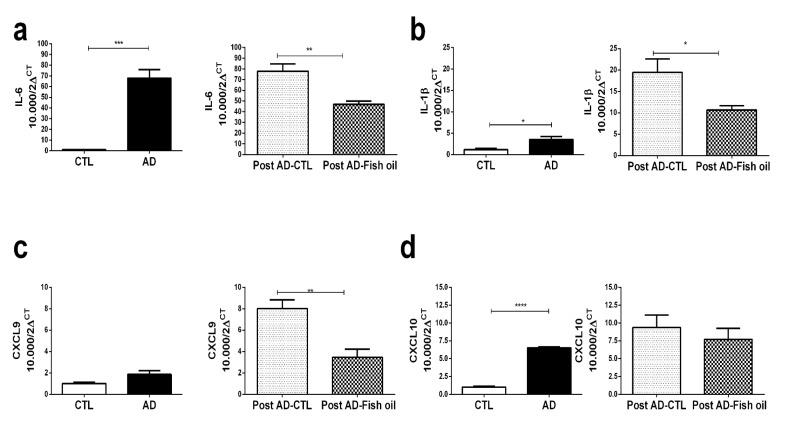
Pro-inflammatory markers: (**a**) IL-6, (**b**) IL-1b, (**c**) CXCL9 and (**d**) CXCL10 in the CTL/AD and Post AD-CTL and Post AD-Fish oil groups (* *p* < 0.05, ** *p* < 0.01, *** *p* < 0.001, **** *p* < 0.0001).

**Figure 3:**
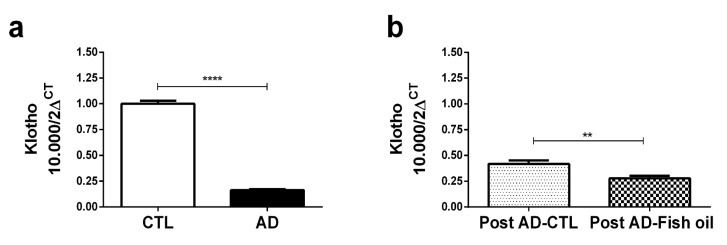
mRNA *Klotho* by PCR: (**a**) CTL/AD and (**b**) Post AD-CTL and Post AD-Fish oil mice (** *p* < 0.01, **** *p* < 0.0001).

**Figure 4:**
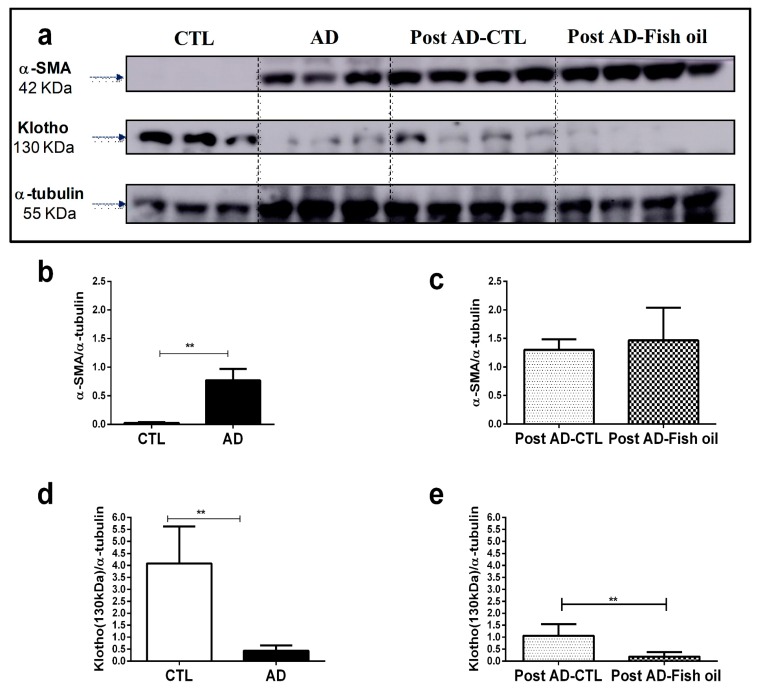
Klotho and alpha smooth muscle actin (α-SMA) expression by Western blot: (**a**) α-SMA and Klotho expression in renal tissue, normalized by α-tubulin in the CTL, AD, Post AD-CTL, and Post AD-Fish oil groups. Relative quantification of α-SMA (**b**,**c**) and Klotho (**d**,**e**) in the CTL, AD, Post AD-CTL and Post AD-Fish oil groups (** *p* < 0.01).

Animals fed with adenine for 10 days exhibited higher mean levels of serum creatinine and urea when compared with the control group (Figure 1b,c). AD mice exhibited significantly higher renal expression of IL-6, IL-1β, and CXCL10 versus CTL (Figure 2a,b,d). Finally, we noted that *Klotho* mRNA expression was at least six-fold lower in the renal tissues of AD mice when compared with CTL (Figure 3a) (0.163 ± 0.01 vs. 1.001 ± 0.02), which was also confirmed by Western blot (Figure 4a,d). In summary, these data indicate that experimental adenine-induced CKD was associated with impaired renal function, increased pro-inflammatory mediators, and decreased *Klotho* expression.

### 3.2. Fish Oil Supplementation Reduces Pro-Inflammatory Markers but Does Not Improve Renal Function 

Fish oil treatment promoted reduction of renal IL-6, IL-1β, and CXCL9 expression (Figure 2a–c). The reduction of CXCL10 by fish oil did not reach statistical significance (Figure 2d). Interestingly, the expression of IL-1β and CXCL9 was even higher in the Post AD-CTL group when compared descriptively with group AD (Figure 2b,c). Such an increase of inflammatory mediators in Post AD-CTL indicated that following the withdrawal of adenine on the 10th day, the process of inflammation continued to be active for the next 7 days during which the animals returned to the standard diet. Present findings suggested that treatment with fish oil exerted some renal anti-inflammatory effects on CKD animals subjected to adenine supplementation. However, the Post AD-Fish oil and Post AD-CTL animals did not differ statistically with respect to renal function (Figure 1d,e).

### 3.3. Fish Oil Supplementation Does Not Revert Progressive Renal Fibrosis 

**Figure 5:**
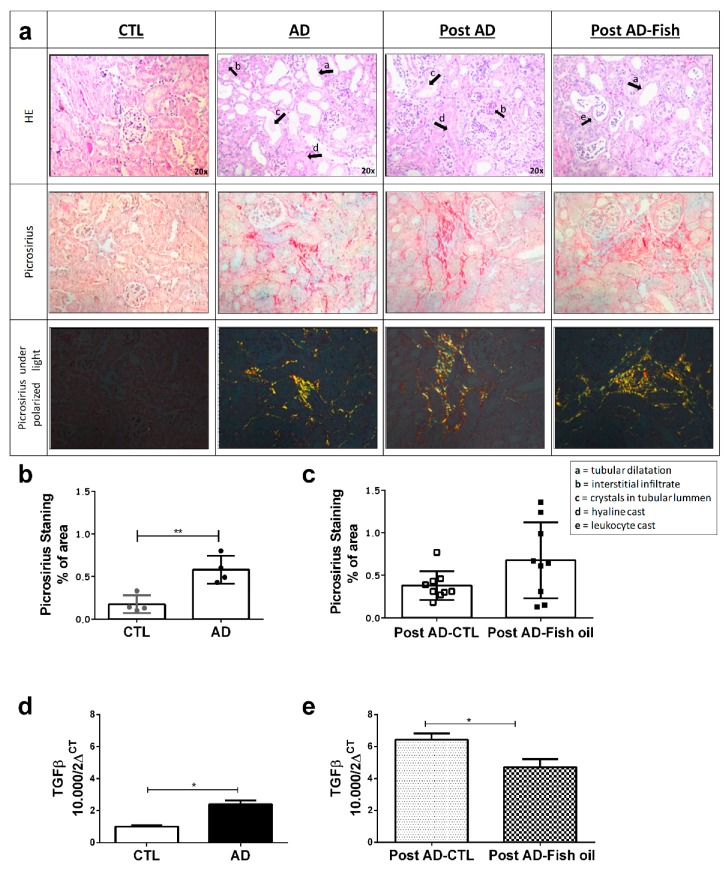
Fibrosis evaluation through picrosirius and transforming growth factor beta (TGFβ): (**a**) Images from Hematoxylin and eosin (HE) stain and picrosirius the kidney samples of CTL/AD and Post AD-CTL and Post AD-Fish oil groups. Graphical quantification of fibrosis observed by picrosirius in (**b**) CTL/AD and (**c**) Post AD-CTL and Post AD-Fish oil groups. TGF-β expression in (**d**) CTL/AD and (**e**) Post AD-CTL and Post AD-Fish oil groups (* *p* < 0.05, ** *p* < 0.01).

The deposition of crystals in tubular lumens from the kidneys of AD mice, coupled with tubular dilatation, interstitial infiltrate and hyaline cylindersresulting in tubular damage was confirmed by histomorphological analysis (Figure 5a). Furthermore, renal deposition of types I and III collagens, revealed by Picrosirius staining, were evidenced in AD mice (Figure 5b). In addition, other pro-fibrotic markers such as transforming growth factor beta (TGFβ) expression (Figure 5d) and alpha smooth muscle actin (α-SMA) evaluated by Western blot, were significantly higher in AD mice compared with CTL (Figure 4a,b). These data confirm that mice fed with adenine for 10 days had tubulointerstitial inflammation with progressive fibrosis.

Both Post AD-CTL and Post AD-Fish oil mice showed the same degree of renal tissue injury as observed in AD mice. (Figure 5a). Similarly, interstitial deposition of types I and III collagen was not statistically different between Post AD-Fish oil and Post AD-CTL mice (Figure 5c). Although TGFβ expression was reduced in Post AD-Fish oil versus Post AD-CTL mice (Figure 5e), we showed that protein expression of α-SMA was not different between both groups (Figure 4a,c). Taken together, these results suggest that fibrosis was not ameliorated by fish oil in this CKD model.

### 3.4. Fish Oil Does Not Restore Renal Klotho

The expression of *Klotho* mRNA (Figure 3b) and transmembrane Klotho expression, shown by Western blot (Figure 4a,e) could not be restored by fish oil treatment; Klotho expression in Post AD-Fish oil was even slightly lower than in Post AD-CTL mice. Nevertheless, this difference did not seem to yield an important biological effect, given that some consequences such as impact on renal function and magnitude of fibrosis observed in both groups were quite similar. 

## 4. Discussion

Several factors contribute to downregulate Klotho expression in CKD, including uremic toxins, vitamin D deficiency, phosphate overload, activation of RAAS, oxidative stress, and inflammation [4,5]. In the same way, experimental studies have shown that exogenous Klotho supplementation or its transgenic overexpression attenuates renal injury [4,13]. In various mice models of inflammatory disease, renal Klotho expression is suppressed [6,14,15,16] and Klotho possesses anti-inflammatory properties as well [17,18]. Given the potential anti-inflammatory effects of omega-3 fatty acids, largely shown in vitro and in vivo [19,20], we hypothesized that fish oil could restore the downregulation of Klotho in an adenine-induced CKD mice model. We found that fish oil supplementation reduced intra-renal inflammation but was not enough to ameliorate renal function and interstitial fibrosis nor retrieved kidney Klotho expression. The rationale for choosing the experimental model of adenine-induced tubulointerstitial nephritis [21] relied on the characteristic features of progressive renal dysfunction and interstitial fibrosis of this model [22] coupled with an intense local tissue inflammation with high expression of pro-inflammatory cytokines [12] which ultimately leads to progression of the disease. The model resembles the adenine phosphoribosyl transferase (APRT) deficiency, a rare human monogenic disease in which adenine cannot be salvaged to adenosine monophosphate, but is catabolized instead to 2,8-DHA in which crystals deposition leads to irreversible renal failure [23,24]. In the present study, aiming to determine the therapeutic rather than the preventive effects of fish oil on the amelioration of the renal inflammatory damage, adenine feeding was withdrawn at the 10th day, when according to our experimental design, the animals were either returned to a standard diet or switched to the fish oil-supplemented one. In the first set of experiments, we found that compared to controls, adenine-fed animals exhibited significantly higher renal expressions of inflammatory markers such as IL-6 and IL-1β, and of CXCL10 (a chemokine responsible for leucocyte recruitment), as well as increased renal tissue expressions of α-SMA (indicating myofibroblast deposition), and of TGF-β (a surrogate marker of fibrosis). Histomorphological analyses confirmed the presence of crystals in tubular lumen, tubular dilatation, interstitial infiltration, and leukocyte casts. Fibrosis was clearly evident by picrosirius staining as well. All these findings agreed well with other studies employing the adenine TIN mouse model [12,22,25]. As might be expected, renal function was also reduced in adenine-fed mice groups, as evidenced by the rises in serum urea and creatinine, in agreement with previous reports [12,21,22,25]. Although the elevation of serum creatinine level in this model has been described in a time-dependent manner, this parameter is already significantly higher after 7 days following the initiation of adenine feeding [22]. Moreover, previous data from our group have shown that the inflammatory process and enhanced cellular infiltration accompanied by collagen deposition leading to a progressive renal dysfunction is readily installed at 10 days after adenine supplementation [12]. In view of the presence of CKD [4,26,27], inflammation [15,16], or both, current experiments disclosed a marked reduction in renal Klotho expression in these adenine-fed animals, corroborating previously published data who showed extremely low levels of serum and renal Klotho after 4 and 6 weeks of adenine feeding [25,28]. Of note, our findings revealed an even earlier Klotho loss, at 10 days. A precocious Klotho deficiency has been described at the earliest stage of CKD (stage 1) when Glomerular Filtration Rate (GFR) is still normal [29]. Animal experiments have demonstrated that EPA and DHA provide benefits in a range of models of inflammatory conditions [30]. Nevertheless, the results of human clinical trials on prevention of inflammation-driven diseases using fish oil have been heterogeneous, with no overall clear evidence of efficacy [31]. With respect to kidney function, a small beneficial effect of an additional amount of 400 mg EPA–DHA per day on kidney function in patients with a history of myocardial infarction and a low habitual EPA–DHA intake has been observed [8]. However, no beneficial effect on inflammation markers such as high-sensitivity C-reactive protein (hsCRP) was further obtained [32]. Dose-dependent actions of marine omega−3 Polyunsaturated Fatty Acids (PUFAs) on inflammatory responses have not been well described, but it appears that a dose of at least 2 g per day is necessary to achieve an anti-inflammatory effect, an unlikely quantity to obtain from the diet [31]. Although other randomized controlled trials employing higher doses of omega-3 fatty acids also did not show a significant effect on circulating parameters of inflammation [33,34], an effect on intra-renal inflammation could not be ruled out by clinical studies. 

In the present study, fish oil promoted a reduction in renal expression of IL-6, IL-1β, and CXCL9, reflecting, at least in part, diminished intra-renal inflammation. This is in accordance with experimental data in rodents dealing with other types of renal injuries such as tacrolimus-induced nephrotoxicity and polycystic kidney disease [35,36]. Despite of the decreased inflammation markers, we observed no restoration of renal expression of Klotho in the fish oil group. As reduced Klotho expression level in the kidney may sensitize the kidneys to injury and aggravation of renal interstitial fibrosis [37,38], hence accelerating renal disease progression, a vicious cycle may ensue. 

Several other reasons might also explain why renal Klotho expression could not be restored. Given the persistence of loss of renal function in our model, irrespective of the fish oil administration, Klotho deficiency is expected, since the kidneys produce/release α-Klotho into the circulation and help clearing it [27]. Moreover, in the CKD setting, over production of reactive oxygen species [39], elevation of uremic toxins [40], high serum phosphate/excess FGF23 [41], and low serum 1,25-dihydroxy vitamin D3 (1,25 Vit D3) are expected to further suppress Klotho production [37,42,43]. Unfortunately, parameters of mineral metabolism such as phosphate, FGF23, and 1,25 Vit D3 have not been measured in the present study.

Other factors which could account for by renal Klotho deficiency in some circumstances depend on its epigenetic modulation. Methylation of the *Klotho* gene promoter, which has been shown to reduce its activity up to 40%, may inhibit Klotho gene expression in CKD [39,44,45] and TGF-β is known to induce global changes in DNA methylation [46]. Accordingly, demethylation of *Klotho* gene promoter remarkably reversed renal Klotho deficiency and reduced renal fibrosis [25]. The hyperacetylation of histone in the *Klotho* promoter also may contribute to Klotho underexpression [6]. In summary, decreased Klotho expression may result from an epigenetic response to inflammation inside or outside the kidney [47]. The possibility of a time delay in the response to local inflammation in our model cannot be ruled out. One could argue that a persistent uremic environment has not been fully accomplished in 10 days, but an irreversible renal failure after 2 weeks has been achieved in rats [48]. Given that the progression of renal failure is faster in mice, and that the current histomorphometric results obtained 7 days after the withdrawal of adenine feeding (17th day from baseline) showed a still important interstitial fibrosis, indeed suggest that the latter might have been responsible for the lack of Klotho restoration.

The present study had several limitations as well as strengths. The first concerns with the length of adenine administration/fish oil treatment and the eventual concomitancy of both supplementations. Had fish oil treatment been simultaneously administered with the adenine-supplemented diet, no inflammation or a shorter period of inflammation could have occurred, hence preventing the progression of fibrosis and loss of renal function. In this scenario, Klotho reduction could have even been averted. However, as discussed above, we aimed to determine the therapeutic rather than the preventive effects of fish oil on inflammation, given that in the clinical practice, the exact moment of initiation of a renal insult cannot be predicted. Second, higher doses of fish oil to achieve a more potent anti-inflammatory effect could have been employed. Nevertheless, few dose finding experimental and clinical studies have been performed to help determine optimal dose-response effects. A third limitation of the present study was that adhesion molecule expression and leucocyte chemotaxis (CXCL8 and MCP1) have not been currently determined in our model to more properly assess both the magnitude and recovery of inflammation. The lack of measurement of parameters of mineral metabolism such as phosphate, FGF23, and 1,25 Vit D3, which are involved in Klotho regulation, compromised a further discussion of the absence of Klotho recovery. Finally, we are aware that the current model represents a specific tubulointerstitial insult with no glomerular injury, thus not reflecting all types of CKD.More studies employing different timing and dose of fish oil treatment in this and other models of CKD are still warranted in order to better understand the link between Klotho reduction and inflammation, fibrosis, and renal dysfunction. 

## 5. Conclusions

The present study suggested that fish oil supplementation reduces renal expression of pro-inflammatory markers, but was not able to restore renal function or Klotho expression in an inflammatory model of CKD.
